# Repair of parastomal hernias with the intraperitoneal funnel meshes IPST-R and IPST

**DOI:** 10.1038/s41598-024-69667-y

**Published:** 2024-08-09

**Authors:** Semen Ilgeldiev, Soeren Stoeckel, Axel Dievernich, Madeline Schmidt, Hagen Rudolph, Lutz Mirow

**Affiliations:** 1 Department of General and Visceral Surgery, Klinikum Chemnitz gGmbH, Flemmingstraße 2, 09116 Chemnitz, Germany; 2 Institute of Laboratory Animal Science, RWTH Aachen University Hospital, Aachen, Germany

**Keywords:** Parastomal hernia repair, Chimney technique, IPST, Funnel mesh, PVDF, Outcomes research, Colorectal cancer

## Abstract

The treatment of parastomal hernias (PSH) represents a major challenge in hernia surgery. Various techniques have been reported with different outcomes in terms of complication and recurrence rates. The aim of this study is to share our initial experience with the implantation of the DynaMesh-IPST-R and -IPST, intraperitoneal funnel meshes made of polyvinylidene fluoride (PVDF). This is a retrospective observational cohort study of patients treated for PSH between March 2019 and April 2023 using the chimney technique with the intraperitoneal funnel meshes IPST-R or IPST. The primary outcome was recurrence and the secondary outcomes were intraoperative and postoperative complications, the latter assessed using the Clavien–Dindo classification. A total of 21 consecutive patients were treated with intraperitoneal PVDF funnel meshes, 17 with IPST-R and 4 with IPST. There were no intraoperative complications. Overall, no complications occurred in 61.9% (n = 12) of the patients. Major postoperative complications (defined as Clavien–Dindo ≥ 3b) were noted in four cases (19.0%). During the mean follow-up period of 21.6 (range 4.8–37.5) months, one patient (4.8%) had a recurrence. In conclusion, for the treatment of parastomal hernias, the implantation of IPST-R or IPST mesh has proven to be efficient, easy to handle, and very safe. In particular, the low recurrence rate of 4.8%, which is in line with the current literature, is convincing. However, a larger number of patients would improve the validity of the results.

## Introduction

The treatment of parastomal hernias (PSH) represents a major challenge in hernia surgery. The occurrence of PSH is the most common complication after the creation of a permanent stoma. The incidence is estimated to be over 30% after one year, 40% after two years and 50% and more with longer follow-up^1^. There are various surgical options for the treatment of PSH, but complication and recurrence rates are usually unsatisfactory. Different meta-analyses report recurrence rates ranging from 3.5 to 27.9%^2–5^. Patients with a permanent stoma often have a long medical history with tumour diseases and reduced general health. Furthermore, the therapy of PSH is generally more complex compared to other types of hernia^6^. Accordingly, the postoperative complication rate is also high, ranging between 20 and 60%^7–9^. There are various open and laparoscopic treatment options, but there is still no scientific consensus on a standard treatment. Due to the high risk of recurrence, the European Hernia Society strongly recommends not to perform suture repair for elective PSH surgery^1^. Techniques using mesh are described in the literature as good treatment options concerning complication and recurrence rates^1–3^. Mesh-based repairs can be performed open or minimally invasive, with the mesh placed either intraperitoneally or extraperitoneally in an onlay, sublay (retromuscular) or preperitoneal position. The treatment of PSH with mesh in the onlay position cannot be recommended due to insufficient data^10,11^. Intraperitoneal onlay mesh (IPOM) techniques are among the techniques with the best results. IPOM techniques include the keyhole technique with flat incised mesh, the Sugarbaker technique with lateralisation of the stoma loop, sandwich technique, in which the keyhole and Sugarbaker techniques are combined, and the chimney technique with funnel meshes^12^. The chimney technique is a newer approach that has shown promising results in initial studies^13,14^.

The IPST-R and IPST mesh belong to the group of funnel meshes and have been implanted at Chemnitz Hospital since 2019; the first experiences and results are reflected in this study.

## Methods

We conducted a retrospective observational cohort study of 21 consecutive patients with parastomal hernia (PSH) treated with intraperitoneal funnel mesh DynaMesh-IPST-R or -IPST made of polyvinylidene fluoride (PVDF) between March 2019 and April 2023. As part of the preoperative diagnostics, a CT scan of the entire abdomen was performed in all patients.

**Figure 1:**
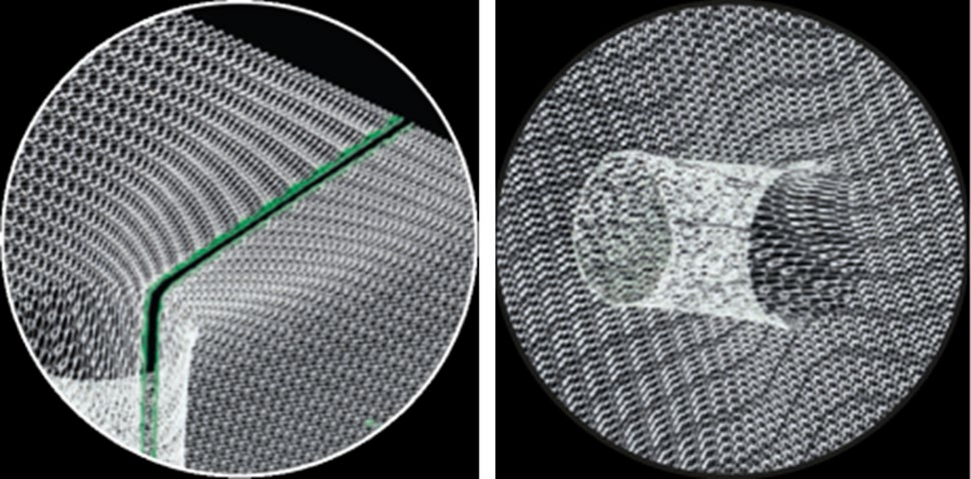
PVDF funnel meshes used: DynaMesh-IPST-R with prefabricated slit and reinforced selvedges with green marking on the left and DynaMesh-IPST without slit on the right.

The DynaMesh-IPST mesh (FEG Textiltechnik, Germany), hereinafter referred to as IPST mesh, is a 3-dimensional mesh with a funnel (diameter = 2 cm, length = 4 cm) without a slit. It was originally developed for the prophylaxis of PSH. In the treatment of PSH, the mesh is either used in a hybrid approach^14–17^, whereby the integrity of the mesh structure is maintained, or it is manually incised and sewn back together after application (not recommended). To maintain the integrity of the mesh structure, the DynaMesh-IPST-R mesh (FEG Textiltechnik, Germany) is available with a prefabricated slit with reinforced selvedges and a slightly larger funnel diameter (3 cm) to achieve sufficient overlap (Fig. 1).

The primary study outcome was the recurrence rate. Secondary outcomes were intraoperative and postoperative complications. For data collection, patient records from the first and all subsequent visits were used (SAP-ERP 6.0/EHP8/Netweaver 7.5). Postoperatively, patients presented in the outpatient clinic for follow-up and aftercare. At intervals of 90 days, 12 and 24 months, the patients received a questionnaire. In addition, all patients were recorded in the Herniamed registry. Patients with an oncological underlying disease are also regularly examined in special outpatient clinics. All mentioned information sources were used to determine complications and recurrences. The Clavien–Dindo classification^18^ was used to evaluate postoperative complications. Statistical analysis was performed using Statistical Package for Social Sciences software (SPSS v27, IBM, USA).

### Information on the surgical technique used

Patients are placed in supine position. In case of a PSH in a terminal, descending stoma, a mini-laparotomy (length 1 cm) is first performed in the area of the right mid-abdomen along the anterior axillary line. A skin incision is made, followed by dissection through the subcutaneous tissue. First the fibres of the external oblique muscle, then the internal oblique muscle and the transverse abdominal muscle are identified. These muscle layers are held apart along their fibre orientation with Langenbeck hooks. The parietal peritoneum is opened under direct vision. The optic trocar is inserted into the abdominal cavity using a guide rod. Before inspecting the entire abdomen, a capnoperitoneum with a pressure of 14 mmHg is created. An additional 5 mm trocar is placed under vision in the area of the right lower abdomen along the middle scapular line. Another 5 mm trocar is introduced into the epigastrium, on the left side of the falciform ligament of the liver.

In case of a PSH following a terminal ileostomy or in the area of the ileum conduit, the optic trocar is placed along the anterior axillary line in the area of the left mid-abdomen. Two additional 5 mm trocars are also positioned along the anterior axillary line, in the area of the left upper and lower abdomen.

**Figure 2:**
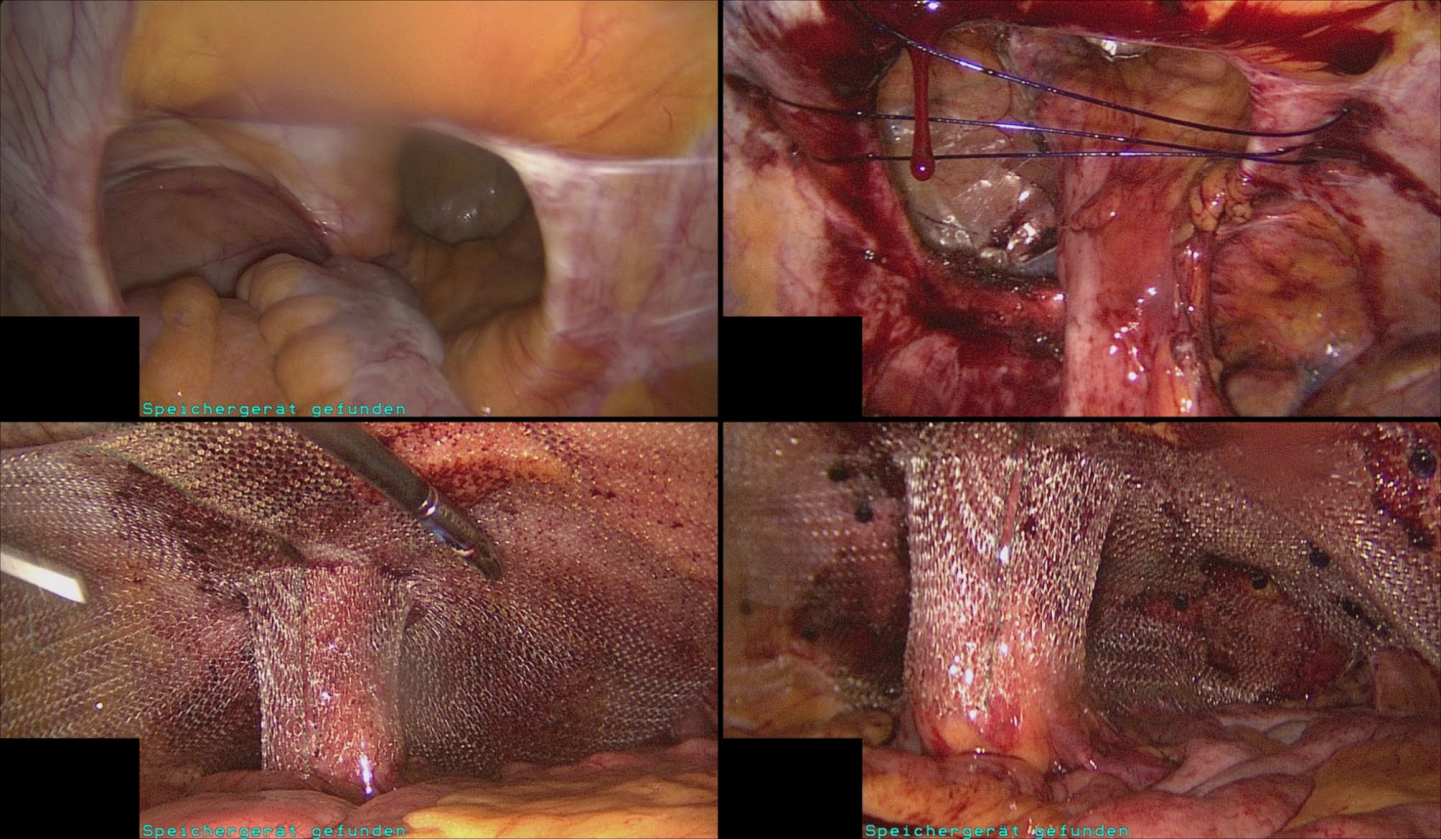
Intrabdominal view of the treatment of a parastomal hernia with an IPST-R funnel mesh from FEG Textiltechnik. From top left to bottom right: parastomal hernia defect, closure of the defect, positioning of the mesh, view after fixation with SorbaFix spiral tacks in double-crown technique, before funnel closure.

After insertion of the trocars, adhesiolysis is usually performed due to the patient’s pre-existing conditions. Subsequently, the fascia is closed with transfascial sutures (using the Endoclose device) with PDS 1-0. When closing the fascia, care is taken to ensure that there is no kinking of the bowel, as this can lead to an ileus/obstruction later on. Then the PVDF funnel mesh is introduced into the abdominal cavity and positioned around the stoma Fig. 2. The mesh is fixed to the ventral abdominal wall in double-crown technique using SorbaFix spiral tacks. The overlapping selvedges of the funnel are secured with a V-Lock suture. During this fixation, the mesentery is also included to prevent stoma prolapse.

### Ethical approval and consent of participants

All experiments on human subjects in this study were conducted in accordance with the relevant guidelines and regulations. The study was approved by the responsible ethics committee, the Saxon State Medical Association. The approval number is EK-BR-18/20-1.

Written informed consent was obtained from all participants before the start of the study. These consent forms confirm that the participants were fully informed about the nature, purpose and potential risks of the study and participated voluntarily. All methods were conducted in accordance with the ethical standards of the Declaration of Helsinki and institutional guidelines.

## Results

The demographic details and parastomal hernia (PSH) characteristics are listed in Table 1. The patients included 7 women (33.3%) and 14 men (66.7%) with a median age of 72 (range 45–89) years and a median body mass index of 28.4 (range 19.5–48.8) kg/m^2^. The most common indication for the first operation was a cancer (61.9%, n = 13). 17 patients (81.0%) were treated for a PSH after a colostomy, three (14.3%) after an ileostomy, and one (4.8%) after a urostomy. An IPST-R mesh was used in 17 cases (81.0%) and an IPST mesh in 4 cases (19.0%).

**Table 1:** Demographic details and characteristics of parastomal hernias.

	*n* = 21	[%] or range
Male/female	14/7	66.7/33.3
Age*, median	72	45–89
BMI, median	28.4	19.5–48.8
ASA score		
2	12	57.1
3	9	42.9
Indication for the first surgery		
Rectal cancer	9	42.9
Colon cancer	2	9.5
Anal cancer	1	4.8
Bladder cancer	1	4.8
Sigmoid diverticulitis	3	14.3
Colitis ulcerosa	2	9.5
Ischemic colonic perforation	1	4.8
Rectal perforation	1	4.8
Small intestinal-vaginal fistula	1	4.8
Stoma type		
Colostomy	17	81.0
Ileostomy	3	14.3
Urostomy	1	4.8
Time* to *PSH*, mean (range)	1.8	0.4–2.5

*In years, *BMI* Body Maxx Index in kg/m^2^, *ASA* American Society of Anesthesiologists, *PSH* Parastomal Hernia.

The postoperative complications assessed according to Clavien–Dindo are listed in Table 2. Overall, there were two grade 1 complications (9.5%; one hematoma, one pain), two grade 2 complications (9.5%; one ileus in a patient with multiple previous surgeries, one obstipation), three grade 3b complications (14.3%; one incisional hernia, one enterocutaneous fistula related to mesh adhesions and pre-existing conditions, one ileus caused by a recurrence 5 days postoperatively due to a technical error during implantation), and one grade 4b complication (4.8%; a cardiopulmonary shock with reanimation and ventilation). No postoperative complication occurred in 13 patients (61.9%).

**Table 2:** Postoperative complications, classified according to Clavien–Dindo.

Clavien–Dindo	1	2	3b	4b
Cardiopulmonary shock	0	0	0	1
Incisional hernia	0	0	1	0
Fistula	0	0	1	0
Ileus	0	1	1	0
Obstipation	0	1	0	0
Hematoma	1	0	0	0
Pain	1	0	0	0
Total (%),* n* = 21	2 (9.5%)	2 (9.5%)	3 (14.3%)	1 (4.8%)

After a mean follow-up period of 21.6 (range 4.8–37.5) months, one patient (4.8%) treated with IPST-R mesh had a recurrence.

## Discussion

The results of this study show that the majority of patients experienced no complications and the recurrence rate was low. This is consistent with the literature, which describes mesh repair techniques as promising treatment options in terms of complication and recurrence rates. The recurrence rate of 4.8% (only one patient) at a mean follow-up of 21.6 (range 4.8–37.5) months is consistent with the results of other studies conducted with IPST mesh. For example, Cartes and Bustos-Jiménez et al. reported a recurrence rate of 9.3% after a mean follow-up of 33 months^19,20^. The cohort consisted of 75 patients, 48 of whom were treated for PSH after an end colostomy, 6 after an end ileostomy and 12 after an urostomy. Tully et al. reported a recurrence rate of 7.4% after a median follow-up of 29 months in 27 patients treated for PSH after urostomy^21^. Other studies conducted with IPST mesh reported recurrence rates between 0 and 15% with mean/median follow-up ranging from 13.5 to 49.5 months^9,13–15,22^.

Overall, the results achieved with the chimney technique using funnel meshes compare favourably with the recurrence rates reported in various meta-analyses for other surgical techniques^2–5^. The results are encouraging, especially considering that patients with permanent stomas often have a long history of tumour disease and poor general health, which can lead to high complication rates^7–9^.

An important aspect to consider is the limited sample size of this study. With only 21 patients, it is difficult to draw universally valid conclusions. However, the results are strengthened when considering the results of the other studies with IPST mesh mentioned above. Nevertheless, future studies with larger patient groups and possibly randomized controlled study designs should help to further strengthen the validity of these results.

## Conclusion

This study has shown that the treatment of parastomal hernias with the intraperitoneal funnel meshes IPST-R and IPST is efficient and very safe. The meshes were easy to handle. The recurrence rate with 4.8% was very low and is in line with the current literature. The majority of patients had no complications. However, it should be emphasized that the limited sample size of our study restricts a comprehensive evaluation. Future studies with larger patient populations and possibly randomized controlled trial designs should strengthen the validity of these results.

## Data Availability

The datasets generated and/or analysed during the current study are not publicly available because the patients have not consented to the publication and forwarding of the data in their declaration of consent, but are available from the corresponding author on reasonable request.
